# Transition from open surgery to ultrasound-guided minimally invasive surgery for insertional Achilles tendinopathy: similar patient-reported outcomes and substantially lower treatment costs

**DOI:** 10.1186/s12891-026-10631-4

**Published:** 2026-10-21

**Authors:** Louise Lindén, Håkan Alfredson, Maziar Mohaddes, Bjorn E Rosengren

**Affiliations:** 1 Orthopaedics, Department of Clinical Sciences, Lund University, Malmö, Sweden; 2 Department of Orthopaedics Hässleholm, Skåne University Hospital, Hässleholm, Sweden; 3 Department of Community Research and Rehabilitation, Sports Medicine Unit, Umeå University, Umeå, Sweden; 4 Alfredson Tendon Clinic, Ortho Center Skåne, Malmö, Sweden; 5 Department of Orthopaedics, Skåne University Hospital, Malmö, Sweden

**Keywords:** Insertional Achilles tendinopathy, Ultrasound, Surgical treatment, Patient-reported outcome measures, Health economics

## Abstract

**Background:**

Insertional Achilles tendinopathy is commonly treated surgically when conservative management fails. Traditionally, open central-splitting surgery (OCS) has been the standard approach, but minimally invasive ultrasound-guided techniques have recently been introduced. This study compared patient-reported outcomes and treatment costs following a transition in surgical practice from open central-splitting surgery to ultrasound- and colour Doppler–guided minimally invasive wide-awake local anaesthesia no tourniquet (WALANT) surgery.

**Methods:**

This retrospective historical cohort study included fifty-two consecutive patients with insertional Achilles tendinopathy. Twenty patients underwent OCS surgery during 2017–2019, followed by thirty-two patients who underwent US-/CD-guided WALANT surgery during 2020–2022. The primary clinical outcome was the SEFAS score at 12 months. Between-group differences in the 12-month SEFAS scores were analysed using multivariable linear regression adjusted for baseline SEFAS score, age, sex, and BMI. Direct medical costs were estimated from the resource use observed across the two treatment pathways.

**Results:**

The mean SEFAS score improved from 18 at baseline to 40 at 12 months in the US-/CD group and from 23 to 41 in the OCS group. In the adjusted analysis, the mean difference in the 12-month SEFAS was 0.62 points in favour of US-/CD (95% CI, − 3.50 to 4.73). Estimated direct medical costs were $1,432 per patient for US-/CD and $3,343 for OCS, corresponding to an absolute reduction of $1,911 per patient and approximately 50% lower costs with the US-/CD treatment pathway.

**Conclusions:**

Following a transition in surgical practice, US-/CD-guided surgery was associated with similar 12-month patient-reported outcomes and approximately 50% lower direct medical costs compared with OCS surgery. These findings suggest that minimally invasive US-/CD-guided surgery under local anaesthesia may offer a less resource-intensive alternative for the surgical treatment of insertional Achilles tendinopathy.

**Clinical trial number:**

Not applicable.

**Supplementary Information:**

The online version contains supplementary material available at https://doi.org/10.1186/s12891-026-10631-4.

## Background

 Insertional Achilles tendinopathy is a common and often debilitating condition characterised by chronic pain at the Achilles tendon insertion [1–5]. This disorder involves pathological changes affecting the bursae, tendon, and bone and frequently presents with retrocalcaneal bursitis, Haglund deformity, intratendinous calcifications, and bony prominences at the calcaneal insertion site [6–8]. Patients typically present with localised pain and tenderness at the tendon insertion, which is often aggravated by physical activity. Diagnosis is traditionally based on clinical examination supported by radiography and magnetic resonance imaging (MRI). However, ultrasonography (US) has increasingly emerged as a valuable diagnostic tool, offering dynamic imaging as well as Doppler-based visualisation of local blood flow associated with tendon pathology [9].

Previous studies have improved the understanding of the pathophysiology of insertional Achilles tendinopathy. Investigations have demonstrated complex innervation patterns within the affected tissues [6], and the potential role of the plantaris tendon in symptom generation has also been highlighted [10–13]. When conservative treatment, including physiotherapy, fails to relieve symptoms [2, 14–15], surgical treatment may be indicated.

Traditionally, and for severe cases, open surgery using the central splitting approach (OCS) is commonly performed. This technique involves detachment of the Achilles tendon, removal of bony prominences and intratendinous calcifications, and reinsertion of the tendon into the calcaneus [1, 16–18]. Although this procedure has shown reliable clinical outcomes, it involves open surgery and typically requires general or spinal anaesthesia, postoperative immobilisation, and a prolonged rehabilitation period.

More recently, minimally invasive ultrasound-guided techniques have been introduced to treat insertional Achilles tendinopathy [10, 19]. Ultrasound- and colour Doppler–guided (US-/CD) wide-awake local anaesthesia no tourniquet (WALANT) surgery allows targeted treatment of pathological structures under local anaesthesia. This technique permits early weight-bearing without immobilisation and is followed by a structured rehabilitation program. In the orthopaedic department where this study was conducted, surgical practice transitioned from OCS surgery to US-/CD-guided WALANT surgery, with mentorship available during the initial six months of implementation [19]. Similar minimally invasive ultrasound-guided techniques have demonstrated promising results in other tendon disorders [20–21].

With increasing emphasis on value-based healthcare and cost-effectiveness, evaluating the economic impact of surgical treatment strategies has become increasingly important. However, despite the growing use of ultrasound-guided WALANT procedures, direct comparisons of treatment costs and clinical outcomes between these techniques and traditional open surgery for insertional Achilles tendinopathy remain limited.

Therefore, the aim of this study was to evaluate the clinical outcomes and direct treatment costs associated with a transition in surgical practice from OCS surgery to US-/CD-guided surgery. The primary objective was to assess patient-reported outcomes 12 months postoperatively. The secondary objective was to compare the total direct treatment costs of the two surgical methods. The study protocol was designed in accordance with the CHEERS reporting guidelines.

## Methods

### Study design and participants

The study was conducted at a regional orthopaedic department in southern Sweden serving a defined catchment population. This retrospective historical cohort study reflects a transition in surgical practice at the department, with OCS surgery being used during the initial period and US-/CD-guided surgery being used during the subsequent period. Clinical outcomes and resource utilisation were compared between these two sequential cohorts.

Before 2020, patients with insertional Achilles tendinopathy underwent open surgery using the central-splitting approach. From 2020 onwards, the surgical strategy changed, and patients were instead treated using US-/CD-guided surgery.

All US-/CD-guided surgeries were performed by the same foot and ankle surgeon. Patients in both groups were included consecutively. The indication for surgery was persistent insertional pain and functional limitations despite structured conservative treatment.

Inclusion criteria were as follows:

- age ≥ 18 years.
- insertional Achilles tendinopathy diagnosed by a foot and ankle surgeon.
- symptoms lasting longer than six months.
- failure of at least three months of physiotherapist-supervised conservative treatment.
- no previous surgery for the same diagnosis on the same side.
- no history of Achilles tendon rupture.
- no pregnancy or breastfeeding.
- no known rheumatic disease.
- no other significant medical conditions judged by the surgeon to increase surgical risk (e.g., severe obesity, poorly controlled diabetes mellitus, or high-dose corticosteroid therapy).
- willingness to undergo surgery and adhere to postoperative rehabilitation, the ability to provide informed consent and the ability to complete study questionnaires.

Twenty consecutive eligible patients (12 men, 8 women; mean age 53 years) underwent OCS surgery between 2017 and 2019. All eligible OCS patients treated during the study period were included; none were excluded due to missing SEFAS data. Thirty-two consecutive eligible patients (14 men, 18 women; mean age, 58 years) underwent US-/CD-guided surgery between 2020 and 2022.

Patients in both groups fulfilled the eligibility criteria for both surgical techniques.

### Preoperative assessment

All patients initially attended an outpatient consultation with a foot and ankle surgeon.

Before the transition to US-/CD-guided surgery, patients in the OCS group underwent preoperative MRI and attended a second consultation to finalise the surgical plan. A nurse assistant supported the surgeon during these consultations.

Following the transition, patients in the US-/CD-guided group underwent US/CD assessment by an experienced physiotherapist with ultrasound expertise during the initial outpatient consultation. Clinical findings were compared with US/CD findings to identify pathological structures and plan the surgical procedure. Preoperative MRI was not routinely used in the US-/CD-guided group.

### US-/CD guided surgery

US-/CD-guided surgery was developed as a minimally invasive outpatient alternative to open surgery. Immediately before surgery, a second US/CD assessment was performed by the same experienced physiotherapist to confirm the presence and location of pathological structures and to identify the structures selected for surgical treatment. Ultrasound was not used intraoperatively.

The US/CD assessment included the subcutaneous and retrocalcaneal bursae, Achilles and plantaris tendons, intratendinous ossification, posterior calcaneal spurs, and posterosuperior calcaneal prominence (Haglund deformity). Clinical findings, including the location of tenderness on palpation, were compared with US/CD findings to identify the structures to be treated. Degeneration of the distal Achilles tendon alone was not surgically treated. Small partial Achilles tendon tears were left untreated, whereas larger partial tears resulted in postponement of surgery to allow initial conservative healing. A detailed description of the US/CD assessment and surgical decision protocol is provided in Supplementary Appendix 1.

All patients were positioned prone. The surgical procedure was performed by an experienced foot and ankle surgeon under local anaesthesia in a clean-air outpatient operating theatre, without involvement of an anaesthesiologist or inpatient admission. Local anaesthesia consisted of 10 mL xylocaine with adrenaline (xylocaine + adrenaline 10 mg/mL + 5 µg/mL; Aspen, Umhlanga, South Africa) injected subcutaneously and into the bursae and periosteum.

A 5-cm incision was made medially or laterally depending on plantaris tendon involvement. Bursae were excised when localised tenderness corresponded to enlargement of the bursa with increased Doppler activity. Plantaris tendon involvement was considered present when localised tenderness on palpation over the plantaris tendon corresponded to a thickened and tendinopathic appearance on ultrasound. When both findings were present, the distal 5–7 cm of the plantaris tendon was excised. Intratendinous ossification was selectively excised when associated with marked Doppler activity. Posterior calcaneal spurs were resected using a small osteotome and gouge when localised tenderness corresponded to a spur with increased Doppler activity. Resection of the posterosuperior calcaneal prominence was based on intraoperative assessment of mechanical impingement after removal of the retrocalcaneal bursa.

The surgical cavities were irrigated with 5 mL of bupivacaine (Marcaine^®^, 5 mg/mL, Aspen, South Africa), and loose bone fragments were removed. Haemostasis was secured, and the incision was closed using non-resorbable sutures. Soft dressings were applied, and immobilisation was not used.

### OCS surgery

This procedure, originally described by Nunley et al. [3], involves partial detachment of the Achilles tendon, debridement of degenerative tissue and bony prominences, and reinsertion of the tendon using suture anchors.

OCS surgery was performed by one of two experienced foot and ankle surgeons under general anaesthesia in a clean-air main operating theatre, followed by an overnight hospital stay.

All patients were positioned prone. A posterior longitudinal incision was made; the Achilles tendon was split and partially detached; degenerative tendon tissue and the retrocalcaneal bursa were removed; and the posterior calcaneus was resected using an oscillating saw. The Achilles tendon was reinserted using a four-anchor fixation device (Achilles SpeedBridge™ system; Arthrex, Naples, Florida, USA).

Haemostasis was secured, and the incision was closed using non-resorbable sutures. Dressings and a non–weight-bearing cast were applied.

### Postoperative care and follow-up

All patients received postoperative analgesics for three days (paracetamol, diclofenac and oxycontin).

In the US-/CD-guided surgery group, full weight-bearing was permitted after 24 h because the Achilles tendon insertion was preserved and no tendon detachment or reinsertion was performed. Follow-up after US-/CD-guided surgery consisted of the following steps:

- nurse visit at 3–4 days for wound inspection.
- nurse visit at 3 weeks for suture removal.
- surgeon visits at 6 and 12 weeks.
- Patients followed a structured 12-week rehabilitation program.

For patients who underwent OCS surgery, the follow-up began at three weeks, when the cast and sutures were removed. Patients were then transitioned to a weight-bearing walker boot with wedges, with a gradual reduction in plantar flexion during a 10-week period.

Postoperative follow-up in the OCS group was individualised according to clinical needs and progress and did not follow a fixed schedule. The mean number of postoperative follow-up visits was five.

### Outcome measurement

Clinical outcomes were assessed using the Self-Reported Foot and Ankle Score (SEFAS), a validated patient-reported outcome measure consisting of 12 questions with a maximum score of 48. The minimal clinically important difference is five points [22].

Patients in the US-/CD group completed the questionnaire at the outpatient clinic preoperatively and at 12 months postoperatively. In the OCS group, the SEFAS scores were collected through the national quality register Swefoot [23]. Complete baseline and 12-month SEFAS data were available for all included patients in both groups. At the 12-month follow-up, all patients also rated their satisfaction with the treatment outcome on a five-point scale: very satisfied, satisfied, somewhat satisfied, dissatisfied, or very dissatisfied. Satisfaction was recorded in the Swefoot register for the OCS group and on a paper questionnaire for the US-/CD group. VISA-A scores were not available for the historical OCS cohort because VISA-A is not included in the Swefoot register and could therefore not be used for between-group comparisons. The primary clinical outcome was the SEFAS score at 12 months postoperatively.

### Cost measurement

A cost analysis was performed, including costs for medical personnel, radiological examinations, surgical materials, and outpatient and inpatient care.

Personnel costs were obtained from the hospital financial controller based on salary levels, including social security contributions.

Information regarding clinical visits, procedures, and radiological examinations was obtained from the Patient Administrative System.

The primary cost analysis included only direct medical costs. Non-medical personnel costs (such as reception and cleaning staff) were not included. Indirect costs, including productivity loss due to sick leave, were evaluated separately in a scenario-based sensitivity analysis.

For the sensitivity analysis, observed sick leave data were available for all 20 employed patients in the US-/CD group, with a mean duration of 24.8 days (3.54 weeks). Individual sick leave data were not available for the historical OCS cohort. Therefore, OCS sick leave durations of 12, 14, and 16 weeks were evaluated as scenarios, reflecting the approximately 3–4 months of postoperative sick leave typically used in our clinical setting. Productivity loss was estimated at $1,100 per week of sick leave, based on 2022 data from the Swedish Social Insurance Agency [www.forsakringskassan.se/statistik].

A standard outpatient visit with a surgeon was estimated to take 30 min. The mean operating time, total operating room time, and recovery time were recorded for both procedures.

For the operating surgeon, the mean operating time plus 30 min was used to account for additional tasks such as preoperative assessment, preparation, documentation, and patient discharge.

Time for other medical personnel was based on total operating room time.

For the central-splitting procedure, half of the operating room time was allocated to the anaesthesiologist, reflecting the standard local practice in which one anaesthesiologist typically supervises two operating theatres simultaneously.

Cost estimates were based on resource use observed in the study cohorts. Procedure times were available at the individual-patient level, whereas other resource components, including outpatient visits, were based on cohort-level averages. Consequently, total treatment costs were estimated at the cohort level rather than calculated at the individual patient level.

A standard nurse visit (wound care, cast or suture removal) was estimated at 45 min.

Costs were calculated in Swedish kronor (SEK) and converted to US dollars using the 2022 mean exchange rate (USD 1 = SEK 10.12).

### Statistical analysis

Continuous variables were summarised using means and standard deviations, and categorical variables were summarised using numbers and percentages. Within-group changes in the SEFAS from baseline to 12 months were assessed using the Wilcoxon signed-rank test because the paired differences were not normally distributed. Between-group comparisons of continuous baseline characteristics were performed using Welch’s two-sample t test, and categorical variables were compared using Fisher’s exact test.

The primary between-group analysis of the 12-month SEFAS was performed using multivariable linear regression, with the 12-month SEFAS as the dependent variable and the treatment group, baseline SEFAS, age, sex, and BMI as independent variables. OCS was used as the reference group. Model assumptions were assessed by examination of the regression residuals and influential observations. The treatment effect was reported as the adjusted mean difference with a 95% confidence interval.

Total treatment costs were analysed descriptively because complete total costs were not available at the individual patient level. No statistical hypothesis test was therefore performed to compare total treatment costs between groups.

## Results

### Patient characteristics and perioperative data

The ultrasound- and colour Doppler–guided (US-/CD) surgical treatment group included 32 patients (14 men, 18 women; mean age 58 years), whereas the open central-splitting group included 20 patients (12 men, 8 women; mean age 53 years) (Table 1).

**Table 1:** Characteristics of two patient samples with insertional Achilles tendinopathy treated with ultrasound- and colour Doppler-guided surgery or open central splitting surgery

Characteristic	US-/CD (*n* = 32)	OCS (*n* = 20)	*p* value
Age, years, mean (SD)	58.3 (9.5)	53.4 (10.5)	0.094
Female sex, n (%)	18 (56%)	8 (40%)	0.393
BMI, kg/m², mean (SD)	27.4 (3.9)	28.3 (5.2)	0.534
SEFAS preop, mean (SD)	18.4 (6.2)	23.0 (6.1)	0.011

The US-/CD surgeries were performed as outpatient day surgeries. The mean total operating room (OR) time was 82 min (SD 19), of which 50 min (SD 11) was the operating time. All patients followed the planned postoperative protocol, and two patients required additional postoperative dressing changes.

In the open central-splitting (OCS) surgery group, the mean operating time was 69 min (SD 11), and the mean total OR time was 141 min (SD 43). All patients stayed one night in the orthopaedic ward following surgery. According to the standard postoperative protocol, the dressings were not routinely changed before cast removal at three weeks; however, three patients required additional dressing changes.

All patients in both groups attended two scheduled postoperative nurse visits according to the postoperative protocol.

The procedural components performed in the US-/CD group are presented in Table 2.

**Table 2:** Procedural components performed in the ultrasound- and colour Doppler-guided surgery group (*n* = 32). Patients could undergo more than one procedural component; therefore, categories are not mutually exclusive

Procedural component	*n* (%)
Subcutaneous bursectomy	29 (91%)
Retrocalcaneal bursectomy	21 (66%)
Resection of bony spur	20 (63%)
Resection of posterosuperior calcaneal prominence (Haglund deformity)	8 (25%)
Plantaris tendon excision	12 (38%)

No postoperative infections or revision surgeries occurred in either group during the follow-up period.

### Patient-reported outcomes

In the US-/CD group, the mean SEFAS score improved from 18 at baseline to 40 at the 12-month follow-up (*p* < 0.001).

In the OCS group, the mean SEFAS score improved from 23 at baseline to 41 at the 12-month follow-up (*p* < 0.001).

In the adjusted analysis (adjusted for baseline SEFAS, age, sex and BMI), the mean difference in the 12-month SEFAS was 0.62 points in favour of the US-/CD group (95% CI, − 3.50 to 4.73). At 12 months, 29 of 32 patients (91%) in the US-/CD group and 15 of 20 patients (75%) in the OCS group reported being at least somewhat satisfied with the treatment outcome.

### Resource use and treatment costs

Resource use for both surgical treatments is presented in Table 3.

**Table 3:** Direct medical costs and resource use for ultrasound- and colour Doppler-guided surgery (US-/CD) and open central splitting surgery (OCS) treatment pathways

	US-/CD		OCS	
	Personnel cost ($)	Other costs ($)	Personnel cost ($)	Other costs ($)
**Outpatient consultation**	238 (3)	222	494 (7*)	518
**Ultrasonography**		253		
**MRI**				282
**Surgery**	333	Materials: 97	698	Arthrex Speed Bridge: 368
		Anaesthesia: 8		Materials (other): 205
				Anaesthesia: 16
**Inpatient care (one night at ward)**			303	184
**Nurse visits**	60 (2)	222	60 (2)	222
**Total personnel cost**	631		1555	
**Total other costs**		802		1795
**TOTAL COST**	**US-/CD**:	**1432**	**OCS**:	**3343**

*For the OCS group, seven outpatient consultations comprised two preoperative visits and a mean of five postoperative follow-up visits. Postoperative follow-up was individualised according to clinical needs

The numbers in parentheses indicate the number of visits or examinations. The mean number of nurse visits was two in both groups. Apart from the Arthrex Speed Bridge (two were used per patient), other material costs include draping and basic surgical instruments

The largest cost component for both procedures was the surgical intervention itself, primarily reflecting personnel costs.

The US-/CD group also required fewer postoperative follow-up visits (Table 3).

The estimated total direct medical treatment cost was $1,432 per patient for US-/CD-guided surgery and $3,343 per patient for OCS surgery, corresponding to an absolute cost reduction of $1,911 per patient and approximately 50% lower direct medical costs with the US-/CD treatment pathway.

Among the 20 employed patients in the US-/CD group, the mean duration of sick leave was 24.8 days (3.54 weeks). Using an estimated productivity loss of $1,100 per week of sick leave, the estimated total cost, including productivity loss, was $5,329 per patient for the US-/CD group. Because individual sick leave data were unavailable for the OCS group, scenario analyses assuming 12, 14, and 16 weeks of sick leave resulted in estimated total costs ranging from $16,543 to $20,943 per patient.

## Discussion

In this study, a transition in surgical practice at a single institution was used to compare resource utilisation and outcomes between the two surgical methods. We reported that compared with open central-splitting (OCS) surgery, ultrasound- and colour Doppler–guided (US-/CD) surgical treatment for insertional Achilles tendinopathy was associated with approximately 50% lower treatment costs, with similar patient-reported outcomes at 12 months.

The difference in cost was primarily related to differences in the surgical setting and resource utilisation. The OCS surgical method required surgery in the main operating theatre, general anaesthesia, and an overnight hospital stay. In contrast, the US-/CD-guided method was performed as outpatient surgery under local anaesthesia, which resulted in substantially lower operating room utilisation, anaesthesia requirements, and hospitalisation costs. In addition, patients who underwent OCS had more postoperative follow-up visits, further contributing to the cost difference. Thus, the observed cost difference reflects the complete treatment pathway rather than the surgical procedure in isolation.

Some variability may exist regarding the need for an overnight hospital stay. In the department where this study was conducted, overnight hospitalisation remains the routine practice following OCS surgery. However, in other centres, the procedure may be performed as day surgery, which could affect the magnitude of the cost difference. Even when the cost of overnight hospitalisation was excluded, the estimated treatment cost remained higher for OCS surgery ($2,857 per patient) than for US-/CD surgery ($1,432 per patient).

Only direct medical costs were analysed in the primary cost analysis. Indirect costs, such as productivity loss due to sick leave, were not included. Patients in the US-/CD group were allowed full weight-bearing after 24 h and did not require immobilisation. Among the 20 employed patients in this group, the mean duration of sick leave was 24.8 days (median 26.5 days). In many cases, this was related to restrictions on wearing shoes with a heel counter during the first six postoperative weeks to allow adequate wound healing. Comparable individual sick-leave data were not available for the historical OCS cohort.

During the yearly meeting of the Society of Swedish Foot and Ankle Surgeons (SFAS) in Stockholm 2024, an expert panel of 52 Swedish foot and ankle surgeons reported that 35 of 38 respondents used OCS surgery, typically immobilising patients for 6–8 weeks, with an average duration of sick leave of 3–4 months. There was no consensus regarding the need for overnight hospitalisation.

To explore the potential impact of productivity loss, a scenario-based sensitivity analysis was conducted using OCS sick-leave durations of 12, 14, and 16 weeks. Across these scenarios, the estimated total cost, including productivity loss, was $5,329 per patient for the US-/CD treatment pathway and $16,543–$20,943 per patient for the OCS treatment pathway.

Direct costs for both procedures may vary across countries and healthcare systems. To our knowledge, no previous study has reported detailed direct cost data for US-/CD-guided surgery. A study from the United States, however, reported material costs for the OCS surgery ranging from $364 to $448 [24], while another study estimated the cost of a SpeedBridge suture anchor to be $391 [25]. Some cost components, such as surgical materials and clinical visits, may be relatively stable across healthcare systems, whereas personnel and imaging costs may vary substantially. These factors should be considered when interpreting the generalizability of the present findings.

The results of the present study indicate potential economic advantages associated with the minimally invasive outpatient treatment pathway employed. Both surgical methods resulted in significant improvement in the SEFAS scores at 12 months. In the adjusted analysis, the estimated between-group difference in the 12-month SEFAS was small, and the 95% confidence interval remained within the established five-point minimal clinically important difference for the SEFAS. However, the 12-month follow-up period is a limitation. Recurrence rates following US-/CD-guided surgery have not yet been extensively studied. Recurrence was not an outcome in the present study. Reported recurrence rates following the OCS surgery range from 14% at 6 months [26] to 2% at 12 months [27] and 14% at 50 months [28]. Differences in long-term outcomes may therefore influence the overall economic comparison of the procedures and should be evaluated in future studies.

It should also be emphasised that the parameters of this study reflect the clinical protocol used at the orthopaedic department where the study was conducted. Variability in surgical technique and postoperative protocols has been described in other settings and should be taken into account when interpreting the results [29].

The implementation of US-/CD-guided surgery requires appropriate training in both US/CD assessment and surgical technique. In the present study, the US/CD assessment was performed by an experienced physiotherapist with ultrasound expertise, while the surgical procedure was performed by a foot and ankle surgeon. Mentorship was available for approximately six months during implementation; this period was not used to define a learning curve or a point at which proficiency was considered achieved. The US/CD assessment followed a structured approach in which clinical findings were compared with US/CD findings to identify the structures selected for treatment. To facilitate reproducibility, the US/CD assessment and surgical decision protocol used in the present treatment pathway are provided in Supplementary Appendix 1.

Despite the advantages of the minimally invasive approach, OCS surgery may still play a role. Patients with extensive intratendinous degeneration, large bony prominences, or complex anatomical variants may benefit from open surgical exposure. Furthermore, in settings where US-guided techniques are unavailable or experience with the method is limited, the OCS approach remains a reliable and well-established option.

### Limitations

The retrospective historical cohort design and absence of randomisation represent important methodological limitations. Given that the cohorts were treated during sequential time periods, temporal changes in clinical practice may have influenced the comparison. In addition, the US/CD procedures were performed by one surgeon, and the OCS procedures were performed by two surgeons, which may have introduced surgeon-related differences between the cohorts.

The study included a relatively small number of patients, and no a priori power calculation was performed because the sample size was determined by the available consecutive cohorts. The baseline SEFAS differed between the groups; however, the primary analysis was adjusted for the baseline SEFAS, age, sex, and BMI. Comparable data on symptom duration, activity level, pathological severity, and Haglund deformity were not available for both cohorts.

SEFAS data were collected through different pathways in the two cohorts, although complete baseline and 12-month data were available for all included patients, and this represents a potential source of information bias. The follow-up period was limited to 12 months and therefore does not allow assessment of late complications or longer-term outcomes.

Finally, the economic analysis was based on resource use observed in a single healthcare setting. Individual sick-leave data were unavailable for the OCS cohort; therefore, the sensitivity analysis relied on assumed OCS sick-leave durations. Differences in healthcare organisation and costs between settings should be considered when interpreting the economic findings.

## Conclusions

Both ultrasound- and colour Doppler (US-/CD)-guided surgical treatment and open central-splitting (OCS) surgical treatment were associated with similar 12-month patient-reported outcomes. However, US-/CD-guided surgical treatment was associated with approximately 50% lower treatment costs. These findings suggest that minimally invasive ultrasound-guided surgery performed under local anaesthesia may offer a less resource-intensive alternative for the surgical management of insertional Achilles tendinopathy.

## Supplementary Information

Below is the link to the electronic supplementary material.

Supplementary Material 1

## Data Availability

The datasets generated and/or analysed during the current study are not publicly available due to the inclusion of potentially identifiable patient information but are available from the corresponding author on reasonable request and subject to applicable ethical and legal requirements.

## References

[1] Furia JP. High-energy extracorporeal shock wave therapy as a treatment for insertional Achilles tendinopathy. Am J Sports Med. 2006;34(5):733–40. 10.1177/0363546505281810 16627628

[2] Jonsson P, Alfredson H, Sunding K, Fahlstrom M, Cook J. New regimen for eccentric calf-muscle training in patients with chronic insertional Achilles tendinopathy: results of a pilot study. Br J Sports Med. 2008;42(9):746–9. 10.1136/bjsm.2007.039545 18184750

[3] Nunley JA, Ruskin G, Horst F. Long-term clinical outcomes following the central incision technique for insertional Achilles tendinopathy. Foot Ankle Int. 2011;32(9):850–5. 10.3113/FAI.2011.0850 22097159

[4] Boffeli TJ, Peterson MC. The Keck and Kelly wedge calcaneal osteotomy for Haglund’s deformity: a technique for reproducible results. J Foot Ankle Surg. 2012 May-Jun;51(3):398–401. 10.1053/j.jfas.2012.03.002. Epub 2012 Mar 23. PMID: 22445185. 22445185

[5] Wiegerinck JI, Kerkhoffs GM, van Sterkenburg MN, Sierevelt IN, van Dijk CN. Treatment for insertional Achilles tendinopathy: a systematic review. Knee Surg Sports Traumatol Arthrosc 2013;21:1345–55. 10.1007/s00167-012-2219-8 23052113

[6] Andersson G, Backman L, Christensen J, Alfredson H. Nerve distributions in insertional Achilles tendinopathy - a comparison of bone, bursae and tendon. Histol Histopathol. 2017;32:263–70. 10.14670/HH-11-790 27320798

[7] Kearney R, Costa ML. Insertional Achilles Tendinopathy Management: A Systematic Review. Foot Ankle Int. 2010;31(8):689–94. 10.3113/FAI.2010.0689 20727317

[8] van Dijk CN, van Sterkenburg MN, Wiegerinck JI, Karlsson J, Maffulli N. Terminology for Achilles tendon related disorders. Knee Surg Sports Traumatol Arthrosc. 2011;19(5):835–41. 10.1007/s00167-010-1374-z PMC3076576 21222102

[9] McGarvey WC, Palumbo RC, Baxter DE, Leibman BD. Insertional Achilles tendinosis: Surgical treatment through a central tendon splitting approach. Foot Ankle Int. 2002;23(1):19–25. 10.1177/107110070202300104 11822688

[10] Alfredson H, Isaksson M. Ultrasound and colour Doppler-guided surgery for Insertional Achilles Tendinopathy-results of a pilot study. OJO. 2014;4(1):7–14.

[11] Alfredson H, Masci L, Spang C. Ultrasound and surgical inspection of plantaris tendon involvement in chronic painful insertional Achilles tendinopathy: a case series. BMJ Open Sport Exerc Med. 2021;7:e000979. 10.1136/bmjsem-2020-000979 PMC7986664 33786196

[12] Maffulli N, Testa V, Capasso G, Sullo A. Calcific insertional Achilles tendinopathy - Reattachment with bone anchors. Am J Sports Med. 2004;32(1):174–82. 10.1177/0363546503258923 14754741

[13] Traina F, Perna F, Ruffilli A, Mazzotti A, Meliconi R, Berti L, Faldini C. Surgical treatment of insertional Achilles Tendinopathy: a systematic review. J Biol Regul Homeost Agents. 2016;30(Suppl 1):131–8. 28002910

[14] Kang S, Thordarson DB, Charlton TP. Insertional Achilles Tendinitis and Haglund’s Deformity. Foot Ankle Int. 2012;33(6):487–91. 10.3113/FAI.2012.0487 22735321

[15] Knobloch K, Yoon U, Vogt PM. Acute and overuse injuries correlated to hours of Training in master running athletes. Foot Ankle Int. 2008;29(7):671–6. 10.3113/FAI.2008.0671 18785416

[16] Mahlfeld K, Kayser R, Mahlfeld A, Grasshoff H, Franke J. Value of ultrasonography asa diagnostic tool of the Achilles tendon. Ultraschall Med. 2001;22(2):87–90. 10.1055/s-2001-12854 11398506

[17] Spang C, Alfredson H, Docking SI, Masci L, Andersson G. The plantaris tendon. A narrative review focusing on anatomical features and clinical importance. Bone Joint J. 2016;98–B:1312–9. 10.1302/0301-620X.98B10.37939 27694583

[18] Ricci AG, Stewart M, Thompson D, Watson BC, Ashmyan R. The Central-Splitting Approach for Achilles Insertional Tendinopathy and Haglund Deformity. JBJS Essent Surg Tech. 2020;10(1):e0035. 10.2106/JBJS.ST.19.00035 . PMID: 32368411; PMCID: PMC7161732. PMC7161732 32368411

[19] Lindén L, Granath M, Hedlund P, Spang C, Alfredson H. Ultrasonography and Doppler-Guided Surgical Treatment for Insertional Achilles Tendinopathy: Results From a Case Series in a Southern Sweden County Hospital. Foot Ankle Orthop. 2023;8(1) 10.1177/24730114231165014. PMC10068980 37021119

[20] Alfredson H, Waldén M, Roberts D, Spang C. Combined Midportion Achilles and Plantaris Tendinopathy: A 1-Year Follow-Up Study after Ultrasound and Colour-Doppler-Guided WALANT Surgery in a Private Setting in Southern Sweden. Med (Kaunas). 2023;59(3):438. 10.3390/medicina59030438 . PMID: 36984438; PMCID: PMC10056337. PMC10056337 36984438

[21] Alfredson H, Roberts D, Spang C, Waldén M. Ultrasound- and Doppler-Guided WALANT Arthroscopic Surgery for Patellar Tendinopathy with Partial Rupture in Elite Athletes-A 2-Year Follow-Up of a Prospective Case Series. Med (Kaunas). 2024;60(4):541. 10.3390/medicina60040541 . PMID: 38674187; PMCID: PMC11051799. PMC11051799 38674187

[22] Cöster M, Karlsson MK, Nilsson JÅ, Carlsson A. Validity, reliability, and responsiveness of a self-reported foot and ankle score (SEFAS). Acta Orthop. 2012;83(2):197–203. 10.3109/17453674.2012.657579 PMC3339537 22313352

[23] Cöster MC, Cöster A, Svensson F, Callréus M, Montgomery F. Swefoot - The Swedish national quality register for foot and ankle surgery. Foot Ankle Surg. 2022;28(8):1404–10. 10.1016/j.fas.2022.07.010 . Epub 2022 Jul 29. PMID: 35933290. 35933290

[24] Wolf J, Fallat L, Coffey M. Outcomes and Material Cost Comparison of Transosseous Versus Suture Anchor Fixation of the Achilles Tendon: A Retrospective Study. J Foot Ankle Surg. 2022;61(1):12–6. 10.1053/j.jfas.2021.05.012 34247969

[25] Devries JG, Scharer BM. Double Row Fixation is More Costly and Does Not Have Clinical Benefit Over Single Anchor for Insertional Achilles Tendon Surgery. J Foot Ankle Surg 2023;18:S1067–2516(23)00007–8. 10.1053/j.jfas.2023.01.001 36737321

[26] Phen HM, Manz WJ, Mignemi D, Greenshields JT, Bariteau JT. Outcomes of Operative Management of Insertional Achilles Tendinopathy in the Young vs. Elder Foot Ankle Orthop. 2020;5(3):2473011420926101. 10.1177/2473011420926101 PMC8564938 35097382

[27] Lugani G, Santandrea A, Mercurio D, Puddu L, Silvestri J, Cortese F. Treatment of Achilles insertional tendinopathy: our surgical procedure and medium-term results. Acta Biomed. 2023;94(2):e2023053. 10.23750/abm.v94i2.13834 PMC10210554 37092637

[28] Hörterer H, Oppelt S, Harrasser N, Gottschalk O, Böcker W, Polzer H, Walther M, Baumbach SF. Recurrence rates for surgically treated insertional Achilles tendinopathy. Arch Orthop Trauma Surg. 2024;144(3):1021–7. 10.1007/s00402-023-05155-8 . Epub 2023 Dec 12. PMID: 38085325; PMCID: PMC10896813. PMC10896813 38085325

[29] Kutzer KM, Morrissette KJ, Wu KA, Krez AN, Hinton ZW, Anastasio AT, Nunley JA, Amendola A. Patient characteristics, postoperative protocols, and surgical outcomes in Haglund’s resection: a single-institution retrospective cohort study. Arch Orthop Trauma Surg. 2025;145(1):241:PMID: 40216619 10.1007/s00402-025-05860-6. 40216619

