# Supplementary material for: Transition from open surgery to ultrasound-guided minimally invasive surgery for insertional Achilles tendinopathy: similar patient-reported outcomes and substantially lower treatment costs
Source: BMC Musculoskelet Disord. 2026 Oct 21;27:913. doi: 10.1186/s12891-026-10631-4 (PMC13700294; doi:10.1186/s12891-026-10631-4)
Supplement: Supplementary file 1 [file 12891_2026_10631_MOESM1_ESM.docx]

**Supplementary Appendix 1**

**Ultrasound and colour Doppler assessment and surgical decision protocol for US-/CD-guided surgery**

**Purpose**
This protocol describes the ultrasound and colour Doppler (US/CD) assessment used for patients undergoing US-/CD-guided wide-awake local anaesthesia no tourniquet (WALANT) surgery for insertional Achilles tendinopathy and how clinical and imaging findings were translated into surgical decision-making.

**Timing of assessment**
US/CD examination was performed during the preoperative clinical evaluation and repeated immediately before surgery. The second examination was used to confirm the presence and location of pathological structures selected for treatment and to mark these structures on the skin. Ultrasound was not used intraoperatively. Preoperative MRI was not routinely used for surgical planning.

**General assessment principle**
Surgical treatment was not based on an abnormal ultrasound finding alone. Clinical findings, including the location of tenderness on palpation, were compared to US/CD findings. For the posterosuperior calcaneal prominence (Haglund deformity), the final decision regarding resection was based on intraoperative assessment of mechanical impingement.

**Subcutaneous and retrocalcaneal bursae**
The bursae were assessed for enlargement and increased Doppler activity. Bursectomy was performed when localized tenderness over the corresponding bursa was combined with enlargement and increased Doppler activity.

**Achilles tendon degeneration**
Tendon degeneration was identified by disruption and disorganization of the normal fibrillar pattern with loss of the normal parallel fibre architecture. Distal Achilles tendon degeneration alone was not treated surgically.

**Partial Achilles tendon tears**
Partial tears were identified as focal hypoechoic or anechoic defects within the tendon. Small partial tears were left untreated. When a larger partial tear was identified, surgery was postponed to allow initial conservative healing and reduce the risk of postoperative complete rupture.

**Plantaris tendon**
Plantaris involvement was considered present when localized tenderness on palpation over the plantaris tendon was combined with a thickened and tendinopathic appearance on ultrasound. When both findings were present, the distal 5–7 cm of the plantaris tendon was excised.

**Intratendinous ossification**
Intratendinous ossification was identified as an echogenic bony structure with acoustic shadowing, and surrounding Doppler activity was assessed. Intratendinous ossification was generally left untreated but was dissected from the tendon and excised when associated with marked Doppler activity.

**Posterior calcaneal spur**
Posterior calcaneal spurs were identified as echogenic bony structures with acoustic shadowing. Resection was performed when localized tenderness was combined with a spur on ultrasound together with increased Doppler activity.

**Posterosuperior calcaneal prominence (Haglund deformity)**
The posterosuperior calcaneal prominence was assessed preoperatively as part of the US/CD examination. The final decision regarding resection was made intraoperatively after removal of the retrocalcaneal bursa. The ankle was moved through flexion and extension while the prominence was manually assessed for mechanical impingement against the surrounding soft tissues. The prominence was only resected when mechanical impingement was present.

**Personnel**
In the present treatment pathway, US/CD assessment was performed by an experienced physiotherapist with ultrasound expertise, while the surgical procedure was performed by a foot and ankle surgeon.

**Table S1. Summary of clinical and US/CD findings and corresponding surgical decisions in the present treatment pathway.**

| **Structure** | **Clinical finding** | **US/CD finding** | **Surgical decision** |
| --- | --- | --- | --- |
| Bursae | Localized tenderness | Enlarged + Doppler activity | Excised |
| Plantaris tendon | Localized tenderness | Thickened/tendinopathic | Distal 5–7 cm excised |
| Achilles tendon degeneration | — | Disorganized fibrillar pattern | No direct treatment |
| Small partial Achilles tendon tear | — | Hypo-/anechoic defect | No direct treatment |
| Larger partial Achilles tendon tear | — | Larger defect | Surgery postponed |
| Intratendinous ossification | — | Echogenic + shadowing ± Doppler | Excised if marked Doppler activity |
| Posterior calcaneal spur | Localized tenderness | Spur + shadowing + Doppler | Resected |
| Posterosuperior calcaneal prominence | — | Prominence | Resected if mechanical impingement was present on intraoperative dynamic assessment |
